# Electronic Memory Support System (eMSS): An Innovative Digital Calendar and Training Program for Older Adults with Mild Cognitive Impairment

**DOI:** 10.1002/alz70858_107207

**Published:** 2025-12-26

**Authors:** Neil W. Thomas, Shekinah McClymont, Fjolla Berbatovci, Meg Schwellnus, Fateme Rajabiyazdi, Octavio A Santos, Adrian Chan, Dona E. Locke, Melanie Chandler, Anne Shandera‐Ochsner, Atul Jaiswal, Chantal Trudel

**Affiliations:** ^1^ AGE‐WELL NIH SAM3, Ottawa, ON, Canada; ^2^ University of Ottawa, Ottawa, ON, Canada; ^3^ Bruyere Health Research Institute, Ottawa, ON, Canada; ^4^ Carleton University, Ottawa, ON, Canada; ^5^ The Ottawa Hospital, Ottawa, ON, Canada; ^6^ Ottawa Hospital Research Institute, Ottawa, ON, Canada; ^7^ Mayo Clinic, Scottsdale, AZ, USA; ^8^ Mayo Clinic, Jacksonville, FL, USA; ^9^ Mayo Clinic Health System, Lacrosse, WI, USA; ^10^ Perley Health, Ottawa, ON, Canada

## Abstract

**Background:**

Individuals with mild cognitive impairment (MCI) experience challenges in maintaining independence in their instrumental activities of daily living (IADLs). The memory support system (MSS) is a paper‐based planner intervention to train individuals with MCI to complete personal goals and IADLs independently. As more individuals use electronic calendar systems, the MSS will need to adapt. This project aimed to develop and test an electronic prototype of the MSS (eMSS).

**Method:**

Drawing on the MSS, an eMSS prototype intended for phone/tablet use was developed in Axure, allowing participants to interact with the planner and its sections (calendar, notes, to do list). The prototype was evaluated with MSS trainers and individuals with MCI and their partners using a computer or tablet over a virtual platform. Participants were asked to go through fictional ‘planning’ scenarios which were assessed using cognitive task analysis (CTA). The scenarios were designed to evaluate overall ease of use, and specifics such as navigation between pages/sections and developing schedules and task lists. During sessions, participants were encouraged to 'think aloud,' providing insight into their eMSS experience.

**Result:**

To date, CTA has been conducted with 6 MSS trainers, and 2 patient/partner dyads that completed the MSS training program. Early results provide insight on strengths, challenges and opportunities to optimize the eMSS. Participants could identify and use key features (e.g., menus) and found the symbols and language consistent. However, participants didn’t notice some features (e.g., interactive arrows to navigate across dates) and requested more ‘cueing’ or ‘interactive prompts.’ Participants would prefer to see all information entered on individual pages (which is possible with the MSS) to support better situation awareness.

**Conclusion:**

To respond to the everyday planning needs of individuals with MCI in a digital age, an eMSS was developed, drawing on a clinically trialed hardcopy version (MSS). Positive feedback from MSS trainers and patients with MCI and their partners suggests promise for the overall design. Improvements for the next iteration include designing cues to help users know actions have been completed or when to attend to important information, as well as providing more fulsome information on pages to support awareness of daily tasks.

